# Accelerated MRI-predicted brain ageing and its associations with cardiometabolic and brain disorders

**DOI:** 10.1038/s41598-020-76518-z

**Published:** 2020-11-17

**Authors:** Arinbjörn Kolbeinsson, Sarah Filippi, Yannis Panagakis, Paul M. Matthews, Paul Elliott, Abbas Dehghan, Ioanna Tzoulaki

**Affiliations:** 1 Department of Epidemiology and Biostatistics, School of Public Health, Imperial College London, St Mary’s Campus, Norfolk Place, London, W2 1PG UK; 2 MRC Centre for Environment and Health, School of Public Health, Imperial College London, London, W2 1PG UK; 3 Department of Mathematics, Imperial College London, London, SW7 2AZ UK; 4 Department of Computing, Imperial College London, London, SW7 2AZ UK; 5 Department of Informatics and Telecommunications, University of Athens, Athens, Greece; 6 Department of Brain Sciences, Burlington Danes Building, Imperial College London, London, W12 0NN UK; 7 UK Dementia Research Institute at Imperial College, Imperial College London, London, UK; 8 National Institute for Health Research, Imperial Biomedical Research Centre, Imperial College London, Exhibition Road, London, SW7 2AZ UK; 9 Health Data Research UK London at Imperial College London, Exhibition Road, London, SW7 2AZ UK; 10 Department of Hygiene and Epidemiology, University of Ioannina Medical School, Ioannina, Greece

**Keywords:** Machine learning, Cognitive ageing, Risk factors

## Abstract

Brain structure in later life reflects both influences of intrinsic aging and those of lifestyle, environment and disease. We developed a deep neural network model trained on brain MRI scans of healthy people to predict “healthy” brain age. Brain regions most informative for the prediction included the cerebellum, hippocampus, amygdala and insular cortex. We then applied this model to data from an independent group of people not stratified for health. A phenome-wide association analysis of over 1,410 traits in the UK Biobank with differences between the predicted and chronological ages for the second group identified significant associations with over 40 traits including diseases (e.g., type I and type II diabetes), disease risk factors (e.g., increased diastolic blood pressure and body mass index), and poorer cognitive function. These observations highlight relationships between brain and systemic health and have implications for understanding contributions of the latter to late life dementia risk.

## Introduction

Chronological age is a major risk factor for poorer physical and mental health and chronic later life neurodegenerative diseases^1–3^. Brain structures and functions show considerable heterogeneity, suggesting that they change at different rates between individuals as a consequence of differences in genotype, environment or lifestyle and disease^4^. We therefore hypothesised that age-related differences between brains relative to changes in a “healthy” normative population may provide an index of disease or disease risk.

A variety of approaches have been used for multi-dimensional modelling of “brain age” from brain MRI images and for assessing associations and differences between modelled brain ages and specific health outcomes, exposures or traits^5^. However, most prior studies have had relatively small sample sizes and have been applied in populations selected for a specific clinical pathology or outcome, as large-scale MRI phenotyping of large general populations has not been performed until recently^6^. Previous work also has relied on linear methods that cannot capture non-linear relationships within the data or “black box” machine learning methods unable to provide information concerning which brain image features were predictive, limiting the interpretability of findings.

More recently, studies have taken advantage of large-scale population-based data including those in UK Biobank and have used neural networks and other advanced methods to analyse MRI imaging data^7–13^. Here we build on these recent studies using a deep convolutional neural network (CNN) with T1-weighted brain MRI data from 21,382 volunteers in the UK Biobank. Our CNN model for predicting brain age was trained and validated on brain images from sub-groups selected for their relative health. We were able to assess the relative contributions of features from different brain regions to identify those that were most informative for the model using permutation importance. We then tested for the potential clinical meaningfulness of differences between modelled and chronological brain age differences by exploring associations of these differences with over 1,400 clinical, lifestyle and environmental characteristics for individuals in a different group of 12,296 of the UK Biobank volunteers that had not been stratified for relative health.

## Results

### Brain age prediction using the optimised neural network

The model-defined and chronological ages were strongly correlated in the training set of healthy individuals (N = 3,067, Pearson correlation = 0.97, mean absolute error = 1.71 years). However, the neural network showed a linear bias for age; individuals older/younger than the cohort average were predicted to be younger/older than they are. Similar bias has been reported and investigated in previous studies^12^. After linearly adjusting the model output for chronological age using data from the training set, the model showed no significant bias on the validation set (N = 3,926). We explored the model’s accuracy on a test set containing only healthy individuals (N = 2,057), on which it achieved a mean absolute error of 2.87 years.

**Figure 1:**
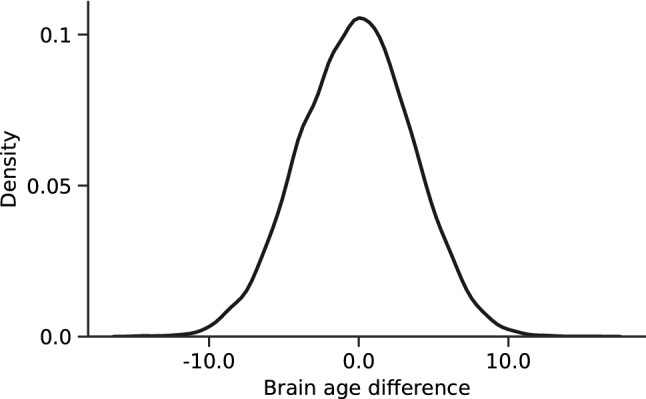
Distribution of brain age differences across the test cohort. Standard deviation is 3.72 years.

**Figure 2:**
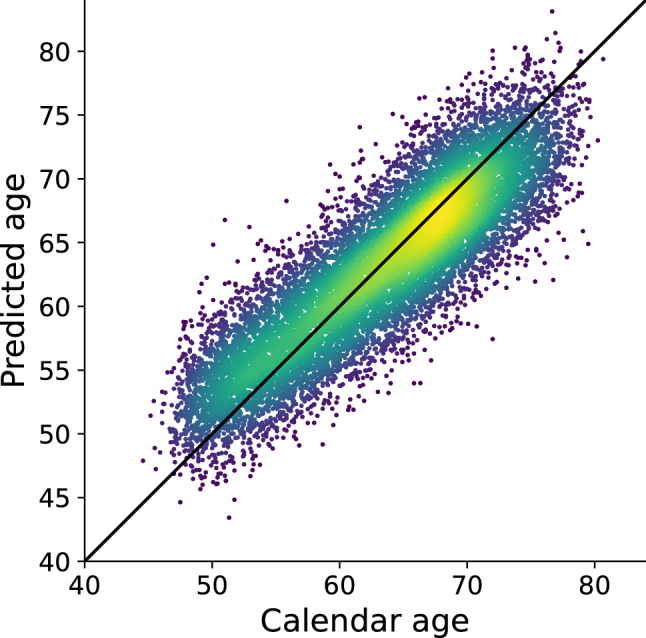
Age predicted by the deep neural network developed here, and linearly adjusted for age using coefficients calculated from the training set, plotted against calendar age for all participants in the test set. The diagonal line is y = x, or a perfect predictor. Colour indicates the density of the scatter with brighter being denser. The Pearson r is 0.82.

We then applied the model to the larger test set who had not been stratified for health (N = 12,196). The model achieved a mean absolute error of 3.42 years and results described a unimodal distribution of differences between predicted and chronological ages (Fig. 1). There was a strong direct relationship (Fig. 2, Pearson correlation coefficient 0.82, *P* = 2.67 × 10^–242^).

### Contributions of different brain regions to brain age predictions

**Figure 3:**
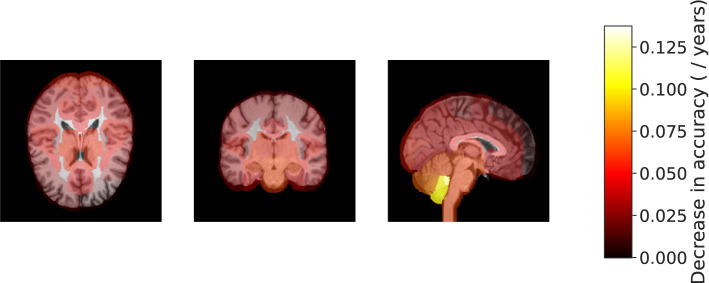
Regions of the brain highlighted by importance on age predictions from T1-weighted brain MRI. Each region (139 total) is overlaid with a constant color representing the decrease in accuracy the results from removing information in that region. Brighter color overlay indicates that a region was more salient to brain age differences as defined by permutation importance.

We attempted to partially explain those image features contributing most to the age prediction model. We assessed differential contributions of brain regions at the level of major white and grey matter regions by serial inference of the model with image regions permuted between individuals. Six brain regions (left cerebellar lobules I-IV and the left crus and vermis, the right hippocampus, left amygdala, and left insular cortex) were found to contribute most to the accuracy of age prediction (Fig. 3 and Supplementary Fig. 1). Removing the information contributions of any of those regions caused the mean average error to increase by more than 0.10 years.

### Phenome-wide association study

**Figure 4:**
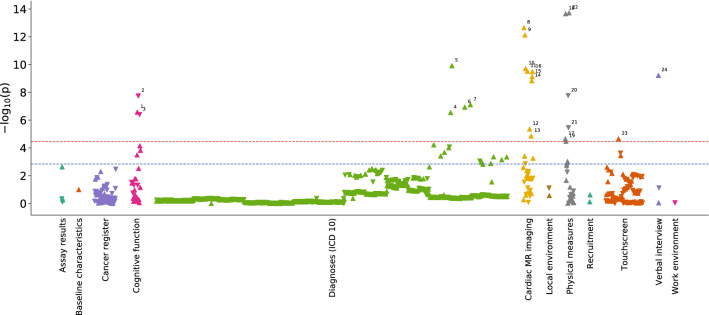
Manhattan type plot showing the significance of association (p-value) between 1,410 UK Biobank traits and brain age difference, coloured by trait category. The Bonferroni-corrected significance threshold is marked by a horizontal red line (p-value = 2.35 × 10^–5^) and the 5% FDR correction threshold with a blue line (p-value = 1.45 × 10^–3^). More details on significant traits is found in Table 1. Trait label 1: Time taken to start entering values in symbol-digit matching test, 2: Number of symbol digit matches made correctly, 3: Number of symbol digit matches attempted, 4: Multiple sclerosis, 5: Essential (primary) hypertension, 6: Diagnoses—secondary ICD10: Type 1 diabetes, 7: Type 2 diabetes, 8: Systolic brachial blood pressure during pulse wave analysis (PWA), 9: Central systolic blood pressure during PWA, 10: Cardiac index during PWA, 11: End systolic pressure during PWA, 12: Stroke volume during PWA, 13: Central augmentation pressure during PWA, 14: Cardiac output during PWA, 15: Central pulse pressure during PWA, 16: Peripheral pulse pressure during PWA, 17: Ventricular rate, 18: Diastolic blood pressure, 19: Body mass index (BMI), 20: Hand grip strength (left), 21: Hand grip strength (right), 22: Systolic blood pressure, 23: Taking insulin, 24: Number of treatments/medications taken.

We then explored the potential meaningfulness of differences between model predicted and chronological ages in the unstratified test population. We explored associations of these differences with more than 1,410 of the International Statistical Classification of Diseases and Related Health Problems (ICD) codes, self-reported clinical conditions and physical, lifestyle and environmental phenotypes. Of these, 24 were found to be significantly associated (corrected P < 2.35 × 10^–5^) with brain age differences (20 direct associations; 4 inverse associations) (Fig. 4 and Table 1).

**Table 1:** Traits that associate with brain age difference, with p-value < 2.35 × 10^–5^ (Bonferroni threshold) . Odds ratios and betas are given per unit standard deviation of brain age difference (3.72 years). PWA = pulse wave analysis.

Categorical and ordered traits	Category	Odds ratio (95% CI)	p-value	Rate of incidence (cases/controls)
Multiple sclerosis	Diagnoses	4.04 (2.37, 6.93)	2.85 × 10^–07^	18/12,278
Type 1 diabetes	Diagnoses	2.39 (1.73, 3.29)	1.17 × 10^–07^	49/12,247
Taking insulin	Touchscreen	2.22 (1.55, 3.14)	2.22 × 10^–05^	36/1633
Type 2 diabetes	Diagnoses	1.42 (1.25, 1.61)	7.77 × 10^–08^	334/11,962
Essential (primary) hypertension	Diagnoses	1.22 (1.15, 1.29)	1.22 × 10^–10^	1820/10,476
Number of treatments/medications taken	Verbal interview	1.13 (1.09, 1.18)	6.02 × 10^–10^	12,294

Continuous traits	Category	Beta (95% CI)	p-value	N samples
Systolic brachial blood pressure during PWA	Heart MRI	0.08 (0.06, 0.10)	2.20 × 10^–13^	10,338
Diastolic blood pressure	Physical measures	0.08 (0.06, 0.10)	2.20 × 10^–14^	11,830
Central systolic blood pressure during PWA	Heart MRI	0.08 (0.06, 0.10)	7.59 × 10^–13^	10,337
Systolic blood pressure	Physical measures	0.07 (0.06, 0.09)	1.91 × 10^–14^	11,830
End systolic pressure during PWA	Heart MRI	0.07 (0.05, 0.09)	3.04 × 10^–10^	10,306
Peripheral pulse pressure during PWA	Heart MRI	0.07 (0.05, 0.09)	3.25 × 10^–10^	10,335
Time taken to enter values in symbol-digit matching test	Cognitive function	0.07 (0.04, 0.09)	2.67 × 10^–07^	6592
Central pulse pressure during PWA	Heart MRI	0.07 (0.04, 0.09)	7.47 × 10^–10^	10,335
Cardiac output during PWA	Heart MRI	0.07 (0.04, 0.09)	1.47 × 10^–09^	10,188
Cardiac index during PWA	Heart MRI	0.07 (0.05, 0.09)	1.94 × 10^–10^	10,046
Stroke volume during PWA	Heart MRI	0.05 (0.03, 0.07)	4.36 × 10^–06^	10,190
Ventricular rate	Physical measures	0.05 (0.03, 0.07)	2.23 × 10^–05^	10,656
Central augmentation pressure during PWA	Heart MRI	0.05 (0.03, 0.07)	1.39 × 10^–05^	10,333
Body mass index	Physical measures	0.04 (0.02, 0.06)	3.33 × 10^–05^	12,260
Hand grip strength (right)	Physical measures	− 0.03 (− 0.05, − 0.02)	3.51 × 10^–06^	12,267
Hand grip strength (left)	Physical measures	− 0.04 (− 0.05, − 0.03)	1.72 × 10^–08^	6581
Number of symbol digit matches attempted	Cognitive function	− 0.07 (− 0.09, − 0.04)	4.08 × 10^–07^	6581
Number of symbol digit matches made correctly	Cognitive function	− 0.07 (− 0.10, − 0.05)	1.82 × 10^–08^	10,338

The diagnoses and traits associated with brain age differences included cardiovascular and metabolic diseases and risk factors, cognitive function and physical strength. For example, there was a four-fold higher risk of having been diagnosed with multiple sclerosis (OR 4.04, 95% CI 2.38–6.93) for each positive SD difference between predicted and chronological age (3.7 years). Measures of blood pressure also showed positive associations with brain age difference including the self-reported diagnosis of hypertension (OR 1.22, 95% CI 1.15–1.29 per SD increase in brain age difference) and measured systolic (beta 0.07, 95% CI 0.06–0.09) and diastolic (beta 0.08, 95% CI 0.06–0.10) blood pressures. Direct associations were found between brain age difference and metabolic traits such as type I (OR 2.39, 95% CI 1.73–3.29) and type II (OR 1.42, 95% CI 1.25–1.61) diabetes and participants taking insulin (OR 2.22, 95% CI 1.55–3.14) (Table 1).

Conversely, individuals with a predicted age younger than their chronological age were found to have greater physical strength, as reflected in hand grip strength (beta − 0.03, 95% CI − 0.05 to − 0.02). Brain age differences were inversely associated with improved performance in tests included in the UK Biobank cognitive battery, including time taken to enter values in a digit-symbol matching test (beta 0.07, 95% CI 0.04–0.09) and the numbers of symbols matched correctly (beta − 0.07, 95% CI − 0.10 to − 0.05).

With a less stringent threshold of 5% FDR (*P* = 1.45 × 10^–3^), 20 additional associations with brain age difference were observed (16 direct associations, 4 inverse associations, Supplementary Table 2). Additional positive associations with brain age difference included having had a depressive episode (OR 3.33, 95% CI 1.85–6.01), history of a prior psychiatric episode (OR 1.97, 95% CI 1.35–2.87) and higher neuroticism score (OR 1.07, 95% CI 1.03–1.12).

### Mendelian randomisation analysis of selected traits

We used Mendelian randomisation to investigate the effects of genetic determinants for a range of traits on brain age differences in order to explore the potential for causality (Supplementary Table 3). The association of a higher genetically determined diastolic blood pressure with higher brain age difference in main and sensitivity regression analyses (inverse variance weighted: beta 0.06, p-value 0.01, weighted median: beta 0.07, p-value 0.02, MR Egger: beta 0.14, p-value 0.05) ( Supplementary Table 3 and Supplementary Fig. 2) provided evidence in support of a causal relationship of blood pressure. The MR-Egger intercept, a measure of pleiotropy that may bias the main inverse variance weighted estimate, did not reach statistical significance, further supporting this conclusion. There was also no evidence for heterogeneity (Cochrane’s Q for inverse variant weighted median: 0.14, Cochrane’s Q for MR Egger: 0.15). By contrast, we did not find evidence for a causal influence of diabetes on brain age differences.

## Discussion

In this large study of 21,382 middle and older aged participants with rich brain MRI data, we developed a deep learning approach to calculate brain age difference with respect to a healthy reference population. Brain age difference should reflect cumulative effects on brain structure associated with effects of environmental, lifestyle and disease exposures, as well as individual differences in genotype. We have approached the “explainability” of this measure by characterising the brain regions whose features made the greatest contributions to brain age difference, which were discovered to be the cerebellum, hippocampus, amygdala and insular cortex. Finally, we conducted an exploration of over 1,400 phenotypes and traits and demonstrated associations between brain age difference and clinically meaningful traits related to cardiovascular, metabolic and brain diseases.

Previous studies have used measures of difference or changes in brain or grey matter volumes over time to provide indirect surrogate measures of the relative rates of brain neuronal volume loss during life^14^. Recent studies use MRI features to predict age-related changes in the brain^9–12,15^ and have applied multivariate methods such as lasso^16^ or independent component analysis^17^, to prospectively derived image features. These studies have described structural and functional changes distinguishing individuals in a dataset ^11^, and revealed that many informative features can be related to brain volume and white-matter microstructure differences^10^. However, many of these previous models were trained on unselected populations that included both people who are healthy and those with disease or disease risk factors. We selected a relatively healthy training population, as also described in two recent studies^10,15^, to fit the model so that positive deviations between predicted and calendar ages can be interpreted as potential signals of disease or disease risk. Given that a hypothesis derived from previous studies is that disease or disease risk factors modify brain aging measures, previous approaches may reduce sensitivity to pathology, although strong influences may be captured by even using heterogeneous training sets or model constructs, e.g., the relationship between elevated blood pressure and greater brain age reported by us and in earlier work^10^. It may be one factor accounting for the heterogeneity of brain aging trajectories elegantly described earlier^11^. For our analyses, we selected a relatively healthy training population to fit the model so that positive deviations between predicted and calendar ages can be interpreted as potential signals of disease or disease risk. Additionally, relationships between brain structures and relative signal intensities (e.g., those reflected in MRI “texture” measures) also change^18^. Unlike simple scalar volume measures, these structural and tissue image texture changes are highly multi-dimensional. Linear models may be relatively insensitive to complex modes of variation or interventions. Our approach uses a deep non-linear CNN that enables capture of complex differences between brain structure and a normative model built from features that are not able to be defined a priori. CNNs achieve state-of-the-art performance on many tasks, including age prediction from brain MRI^8,13,15^. CNNs are prone to overfitting, which likely explains the difference between train and test accuracy observed. This highlights the importance of the completely held-out test set such as has been used here for generalisability and comparisons with other work. 2D CNNs have been used effectively^8^ and trade spatial information for the ability to leverage models pre-trained on natural images. Here, we opted for a 3D CNN that retains and leverages the entire MRI volume.

We also identified regions most important for model predictions using permutation importance, an emerging method for interpreting neural network models on brain MRI ^19^. This highlighted brain regions playing central roles in cognition and memory (hippocampus), emotional regulation and salience (amygdala) and physiological homeostasis (insula). All of these regions have been recognised previously as having a functional role or showing population differences relevant to brain health^20–22^. The importance of the cerebellum for brain age is of particular interest, as relationships between cerebellar pathology and cognitive dysfunction or late life neurodegenerative diseases remain poorly described^23,24^. All other regions did have a smaller, but measurable, impact on model predictions (supplementary Fig. 1). This suggests that the model might have learned a global representation along with more local, region-based patterns. Our observations point to the importance of future work in applying emerging approaches that combine principles from statistics and machine learning for models based on learned features that can be described with greater granularity. Their discovery could contribute to better understanding of the mechanisms underlying relationships between brain structure and disease risk.

Brain structural changes learned by model correlated with clinical diagnoses and phenotypic characteristics, as well as cognitive function, extending prior studies based on different models and using different training sets that also described deviations of predicted ages from chronological ages (similar to the brain age difference metric described here) amongst pathological or “at risk” subgroups of the larger cohort ^8–11,25–29^. Here, individuals with higher brain age difference performed worse in cognitive tests for fluid intelligence, giving support to the index as an informative metric. In terms of diseases, participants with multiple sclerosis had higher brain age differences, revealing changes to brain in addition to those that are age-related. Indeed, multiple sclerosis is associated with macro- and microscopic inflammatory and demyelinating pathology in both white and grey matter^30^ and has previously been associated with increased brain age differences^31,32^. In contrast, we did not observe a statistically significant association between brain age difference and cerebral infarction. This is possibly due to cerebral infarction causing heterogenic brain changes between individuals that are not consistent enough to be associated with a measure across a population. Alternatively, our stringent multiple testing corrections to guard against false positives can potentially cause some true positives to be missed; a limitation of the approach. Analysis showed associations between higher brain age difference and type I and type II diabetes, a finding previously observed in other imaging studies^33^ including analysis on UK biobank^10^ and supported by previously recognised effects of diabetes on brain structure^34,35^. This study also revealed a direct association between brain age differences and vascular disease risk factors, particularly blood pressure. Associations between brain age deviations using distinct approaches to ours but similar data from UK biobank also revealed associated between this phenotype and blood pressure adding internal validity to these results^10^. Although a relationship between hypertension and both cognitive decline and brain atrophy has already been established^35,36^, and prolonged hypertension is recognised to be associated with increased white matter pathology^37,38^ the mechanisms of these associations are not well defined^39^. Physical fitness was associated with lower brain age difference highlighting the association of physical fitness not only to functional^40^, but also to structural changes of the brain.

We examined the potential for causality in the associations between traits and brain age differences. MR analysis demonstrated a likely causal relationship between increased diastolic blood pressure and increased brain age. This implies that reducing diastolic blood pressure would have an impact on the relative brain age, broadly consistent with clinical evidence that reducing or preventing hypertension reduces the risk of strokes^41^. By contrast, our MR analysis did not provide evidence for a causal effect of diabetes on brain age differences, suggesting common, pleotropic factors (pleiotropy) may contribute causally to both, consistent with the possibility that treatments for diabetes mellitus also may have an independent impact on late-life neurodegenerative processes^42^. Future prospective studies relating brain age differences and the incidence of cognitive impairments would add to confidence that the measure could be used as a risk stratification tool for late life cognitive impairments or other brain disorders.

Nonetheless, although we have adopted use of the concept of “brain age” proposed previously, we believe that the term should be used cautiously. Aging is referenced to time since birth, but incorporates concepts of time-dependent intrinsic biological processes and individually specific influences acting on a tissue or person^43^; changes to the brain during the life course are more than solely a consequence of time (age) alone. Our use of a healthy population for training is intended to maximise the interpretability of brain age difference as an index of risk of dysfunction or disease. As noted above, the sensitivity of the approach to factors affecting individuals is limited, as the interpretability analysis is based on population characteristics. Further work could focus on developing descriptions and explanatory hypotheses at an individual level (e.g., using methods such as Shapely Additive Explanations^44^). Another important limitation is the accuracy of region definitions. Although the images were carefully aligned as part of UK Biobank pre-processing, imperfect alignment would cause boundary effects. These were partially mitigated by running the permutation multiple times and averaging the results. However, the relative accuracy of detection of disease or disease risk associations depends on the population sample size and structure; our detections of associations are, for example, impacted by the relatively low prevalence of stroke, multiple sclerosis and diabetes in the population studied. We are making our model openly available for others but need to highlight that it was developed and validated with UK Biobank data; generalisation would require extending training to include new target populations or data acquired using different MRI platforms or sequences. Finally, we analysed the associations between genetic variants and brain age differences in order to perform MR analysis; however, an in-depth investigation of genetic determinants of brain age was beyond the scope of this work and has been covered elsewhere^9,13^. We adopted a hypothesis free approach to investigate a range of phenotypes in relation to brain age differences with adjustment for multiple comparisons. However, different associations had different sample sizes and therefore power to detect associations which should be taken into account when interpreting the associations.

Our results add to a growing literature showing that brain structural differences provide a general marker of systemic health. They suggest that brain age difference may be an index of health or risk of later life metabolic, cardiovascular and brain diseases and functional traits relevant to health. Consistent with conclusions from large cohort treatment studies^41^, our results suggest a direct causal link between higher diastolic blood pressure and brain age difference. With larger populations and further refinement of methods, the approach may help to better define risk factors of late life brain disease. Stratifying people on this (or a similar) index may help identify those who could benefit most from interventions for risk factor reduction for future brain health. Finally, this work further adds to the literature on the potential use of AI as a decision-support tool to enhance the information available from neuroradiological reporting.

## Methods

### Description of UK Biobank and imaging data

UK Biobank is a population-based cohort study of ~ 500,000 participants, who were recruited from the UK general population between 2006 and 2010. At baseline, participants, who were between 40–69 years old, provided blood samples for biochemical tests and genotyping and a wide range of self-reported information and physical measurements, and consented for their data to be linked to Hospital Episode Statistics (HES). Detailed protocols for obtaining the measurements from participants have been described^6^. The UK Biobank resource is open to all bona-fide researchers anywhere in the world, including those funded by academia and industry. An imaging extension to the existing UK Biobank study was initiated in 2016 with plans to scan 100,000 individuals from the cohort by 2022–23. Here we have analysed the interim release of T1-weighted structural brain MRI on 21,382 participants from this imaging sub-study^45^. The images were captured on a 3 T Siemens Skyra scanner (software platform VD13). Each image was single channel with dimensions 182 × 218 × 182 at 1 mm^3^ resolution. Images used here were aligned to the MNI152 template^46^ as part of UK Biobank pre-processing.

### Study design

To set up a supervised machine learning framework, we split the data from 21,382 individuals into four sets: a training (N = 3,067), validation (N = 3,962), healthy test (N = 2,057) and unselected (general) population test (N = 12,296) set. Individuals were divided randomly between the four sets, subject to restrictions on the number of apparently healthy and unhealthy individuals in each set. Healthy participants were defined as those who did not report health conditions or chronic treatments for health conditions and hospital ICD coding available through UK Biobank did not identify chronic disease at the time of, or preceding, baseline assessment. The training set was used to optimise parameters of the neural network and learn relationships between healthy brain structure and age. This allowed the model to learn a representation of the physiological age-related modifications in brains of healthy individuals as a proxy for changes occurring with normal aging processes. Including brains from unhealthy individuals would have forced the model to learn structural changes related to disease and correct its predictions accordingly. The validation set was used to tune and assess the model without exposing it to individuals reserved for the final analysis.

We used two separate hold-out test sets to evaluate model performance. The former containing only healthy individuals to test the model on unseen healthy individuals. The latter test set was used for conducting the phenome-wide association study and included individuals who had not been stratified for health status. See Supplementary Table 1 for descriptive characteristics of each data set.

### Deep neural network for brain age difference modelling

The objective was to determine the brain age difference for an individual, given an MRI of their brain and their chronological age. We defined brain age difference as the difference between an individual’s chronological age and the age predicted by the deep learning model from T1-weighted MRI.

The age prediction model was a convolutional neural network^47,48^ that took as input a T1-weighted structural image with dimensions 182 × 218 × 182 and output the predicted age of the person. Our network used 3D convolutional operations, batch-normalisation^49^ and activations with a rectified linear unit, residual blocks, based on those in residual networks (ResNets)^50^. For a detailed description of the model, please refer to the supplementary methods. Training was run by minimising the mean squared error between the true and predicted values and optimized using Adam^51^. We used 3D convolutions throughout the network to leverage the full structure of the MRI. We built the model in PyTorch^52^ and TensorLy^53^. Models were trained on an NVIDIA P100 GPU.

We used a permutation importance approach^19,54,55^ to analyse the importance of different brain regions by quantifying their contribution to model predictions. The method works by repeatedly permuting a specific feature, in this case a region of the brain, between individuals. Serial repetition across the population develops a model of the distribution of predictions without the region that can be compared with that before data removal. For a detailed description of the approach, please refer to the supplementary methods.

### Statistical analysis

We performed a phenome-wide association study to test for associations between brain age difference and 1,410 phenotypic characteristics measured on UK Biobank participants through clinical assessments, record linkage and health and lifestyle questionnaires. We used the PHEASANT analysis method to perform this agnostic scan as previously described^56^. Briefly, the software uses a series of regression analyses (linear for continuous traits and logistic for binary traits) to associate traits with the exposure of interest (i.e. brain age difference). For each trait, samples with missing values were excluded from that analysis. All analyses were adjusted for age, sex and assessment centre. To account for multiple testing, we used Bonferroni correction (P = 2.35 × 10^–05^). In sensitivity analyses, in order to account for the correlation between the measured phenotypes in UK Biobank we also used a false discovery rate (FDR) of 5% using the Benjamini–Hochberg procedure to account for multiple testing^57^.

### Mendelian randomisation

Causality of associations between brain age differences and traits is difficult to infer. Mendelian randomisation provides a method for assessing the potentially causal nature of some associations^58^. We performed two-sample Mendelian randomisation analysis of selected traits (exposure) that had summary GWAS data available (diastolic and systolic blood pressure, pulse pressure, Alzheimer’s disease and diabetes) to explore the causality of the reported associations. Genetic variants used as instruments were obtained from DIAGRAM 1000G study^59^ for type 2 diabetes, from the International Genomics of Alzheimer's Project^60^ for Alzheimer’s diseases, and from a recent study^61^ for blood pressure. For the latter, we used the allele effects from the International Consortium for Blood Pressure^62^ to avoid bias due to overlapping samples. For the association of the genetic variants with brain age difference, we used the data from UK Biobank. We matched and harmonised the effective allele for each set of instruments with brain age difference and removed the correlated variants using LD clumping (r^2^ < 0.1). We estimated the causal effects using the inverse variance weighted method. Potential pleiotropic effect was detected using heterogeneity tests and sensitivity analysis was done using weighted median and MR Egger regression methods to rule out pleiotropic effects. All analyses were done using the Two Sample MR package^63^.

## Supplementary information

Supplementary Information.
