# Supplementary material for: Accelerated MRI-predicted brain ageing and its associations with cardiometabolic and brain disorders
Source: Sci Rep. 2020 Nov 17;10:19940. doi: 10.1038/s41598-020-76518-z (PMC7672070; doi:10.1038/s41598-020-76518-z)
Supplement: Supplementary file 1 — Supplementary Information. [file 41598_2020_76518_MOESM1_ESM.docx]

**Supplementary material for: Accelerated MRI-predicted brain ageing and its associations with cardiometabolic and brain disorders**

Arinbjörn Kolbeinsson, Sarah Filippi, Yannis Panagakis, Paul M Matthews, Paul Elliott, Abbas Dehghan, Ioanna Tzoulaki

**Supplementary Methods**

**MRI data pre-processing**

The raw MRI voxel intensities have arbitrary units and are not standardised between images. This can cause model training to become unstable since training errors and model parameters will be on different scales. An update that is too small will not change the model parameter values while updates that are too large can cause numerical overflow. To avoid this, we normalised and scaled the data to remove these major inter-image biases; each image was scaled to zero mean and unit standard deviation. In addition, we found that scaling the target variable, participant’s age, by a factor of 0.01 resulted in more stable training behaviour. Without this scaling the model would output predictions only in a limited range that was smaller than the range of the target distribution.

**Brain age difference model**

We defined brain age difference as the difference between an individual’s chronological age and the age predicted by the deep learning model from T1-weighted MRI trained on the healthy aging training cohort. For example, a 50-year-old (chronological age) individual whose brain has the structure similar to a 40-year-old (predicted age), has a brain age difference of -10 years. This divergence could be the result of different exposure to risk factors that cause structural changes to brain, or to genetic differences. The objective was to determine the brain age difference for an individual, given an MRI of their brain and their chronological age.

To this end, we employed supervised learning, a class of machine learning where a mapping function *f* from input data (here, T1-weighted structural images) to an output value (here, the individual’s age) is learned based on training data samples based on a subset of the UK Biobank cohort without major reported diseases. The model (i.e., mapping function) was expressed as a convolutional neural network^[1,2]^, whose parameters are learned by iteratively making predictions and adjusting its parameters based on the distance between the true and predicted outputs, known as error or loss. Our model was expressed as:

age_predicted_ = *f*(X_MRI_)

where age_predicted_ was the age predicted by the neural network *f,* for a given input volume X_MRI_. Given the assumption that

age_predicted_ = age_chronological_ + brain age difference + ε

where brain age difference represents biological differences, unrelated to age, that manifest as changes in brain structure and ε is the model error. Our aim was to disentangle brain age difference from model error ε.

However, estimating brain age difference directly was not possible since we did not have the ‘ground truth’ brain age difference signal. It was conflated with the model error during training. To combat this, we defined our training set to include healthy participants only, in which age was the dominant contribution to time-dependent effects on the brain and in which brain age difference was assumed to be minimal.

**Deep Neural Network for Age Prediction**

The age prediction model was a convolutional neural network that took as input a T1-weighted structural image with dimensions 182 × 218 × 182 and output the predicted age of the person. Our network contained 18 layers split into three processing parts. The first part was an initial sequence of three-dimensional (3D) convolutions, batch-normalisation^[3]^, activation with a rectified linear unit and max-pooling. The 3D convolutions had kernel size 3 × 3 × 3, a stride of one and took as input a single-channel image and output a 64-channel activation tensor. The max-pooling operation down-sampled the tensor with a window size of 3 and a stride of 2. The second part contained four residual blocks, based on those in residual networks (ResNets)^[4]^, where we made use of skip-connections. Each residual block had two sets of 3D convolutions (with a symmetric kernel-size of 3), batch normalisation and non-linear activation with a rectified linear unit. Skip-connections help the parameter gradient updates to back-propagate through the network as they allow information to bypass non-linear layers. Down-sampling through the residual blocks was controlled entirely by striding the convolutions by two voxels, instead of a pooling operation. Each of the four blocks generates a more abstract representation of the structural image that is more compact in spatial dimensions with more capacity in the represented channels. The layers output 64, 128, 265 and 512 channels, respectively.

The final part contained average pooling and a fully-connected layer to reduce the dimensionality to a scalar output. The final block returns an activation tensor of shape 512 × 6 × 7 × 6. This tensor was flattened by fully average-pooling^[5]^ the three spatial dimensions. A final fully-connected layer connects the 512 activations to a scalar output representing the predicted age.

We used 3D convolutions throughout the network to leverage the full structure of the MRI. We built the model in PyTorch^[6]^ and TensorLy^[7]^. All models were trained on a single NVIDIA P100 GPU.

We trained the model end-to-end by minimising the mean squared error between the predicted and calendar age. For this minimisation, we used the Adam optimiser^[8]^ for 80 epochs and mini-batch size of 8 with an initial learning rate of 10^-4^, decaying by a factor of 10 at epochs 25, 50 and 75. To reduce overfitting and improve generalisation to unseen data we applied a weight decay (L2/ridge penalty) of 5*10^-4^ on all parameters during training. We selected these hyperparameters by finding the combination that provided the minimum mean squared error on the validation set via a grid search.

The trained network showed a small bias with age. Similar bias has been reported and investigated in previous studies^[9]^. We corrected for this by fitting a linear adjustment to the predictions on the training set. This increased the mean squared error but was done to keep the bias constant across age ranges.

**Permutation importance for interpretation**

We investigated the importance of different brain regions by quantifying their contribution to model accuracy. 139 brain regions were defined in the UK Biobank MNI152 atlas (available as part of UK Biobank data). The deep and non-linear nature of neural networks makes them difficult to interpret. To alleviate this, we used a permutation importance approach^[10]^. For each brain region, the approach consisted of permuting the images corresponding to that region between individuals (e.g. the right insular cortex of individual A gets moved to individual B, while the same region of individual B gets moved to individual C and so on), predicting the ages of all individuals based on these modified brain MRI and measuring the accuracy of our original model to predict age for all individuals with the switched region. This process was repeated 12 times to estimate the mean and variance of the permutations. No assumptions about statistical dependence are made with a permutation analysis**.** We quantified the loss of accuracy in terms of increased mean absolute error, the same metric we used to train the unperturbed model. For a region that was important for age prediction, a degradation in performance and increased prediction error would result. Conversely, permuting regions that contributed insignificantly to the prediction would result in a smaller drop in accuracy. The method is described logically by the following pseudocode:

| **Permutation importance algorithm**  *model* trained on *train set*  *test set error* **is** mean absolute error (MAE) on *test set*  **for** *region* in *MNI152 atlas* **do**: |
| --- |
| **for** *sample* in *test set* **do**:  **create** modified sample by **masking** *region* **in** *sample* **with** *region* **from** another random sample  **predict age** of *modified sample* using *model*  **compute** MAE on *modified samples*  **region importance** is difference between MAE on *modified samples* and *test set error* |

1 LeCun, Y. *et al.* Backpropagation applied to handwritten zip code recognition. *Neural computation* **1**, 541-551 (1989).

2 Fukushima, K. Neocognitron: A self-organizing neural network model for a mechanism of pattern recognition unaffected by shift in position. *Biological cybernetics* **36**, 193-202 (1980).

3 Ioffe, S. & Szegedy, C. Batch normalization: Accelerating deep network training by reducing internal covariate shift. *arXiv preprint arXiv:1502.03167* (2015).

4 He, K., Zhang, X., Ren, S. & Sun, J. in *Proceedings of the IEEE conference on computer vision and pattern recognition.* 770-778.

5 Lee, C.-Y., Gallagher, P. W. & Tu, Z. in *Artificial intelligence and statistics.* 464-472.

6 Paszke, A. *et al.* PyTorch: An Imperative Style, High-Performance Deep Learning Library. *Advances in Neural Information Processing Systems* **32**, 8024-8035 (2019).

7 Kossaifi, J., Panagakis, Y., Anandkumar, A. & Pantic, M. Tensorly: Tensor learning in python. *The Journal of Machine Learning Research* **20**, 925-930 (2019).

8 Kingma, D. P. & Ba, J. Adam: A method for stochastic optimization. *arXiv preprint arXiv:1412.6980* (2014).

9 Smith, S. M., Vidaurre, D., Alfaro-Almagro, F., Nichols, T. E. & Miller, K. L. Estimation of brain age delta from brain imaging. *NeuroImage* **200**, 528-539 (2019).

10 Breiman, L. Random forests. *Machine learning* **45**, 5-32 (2001).

**
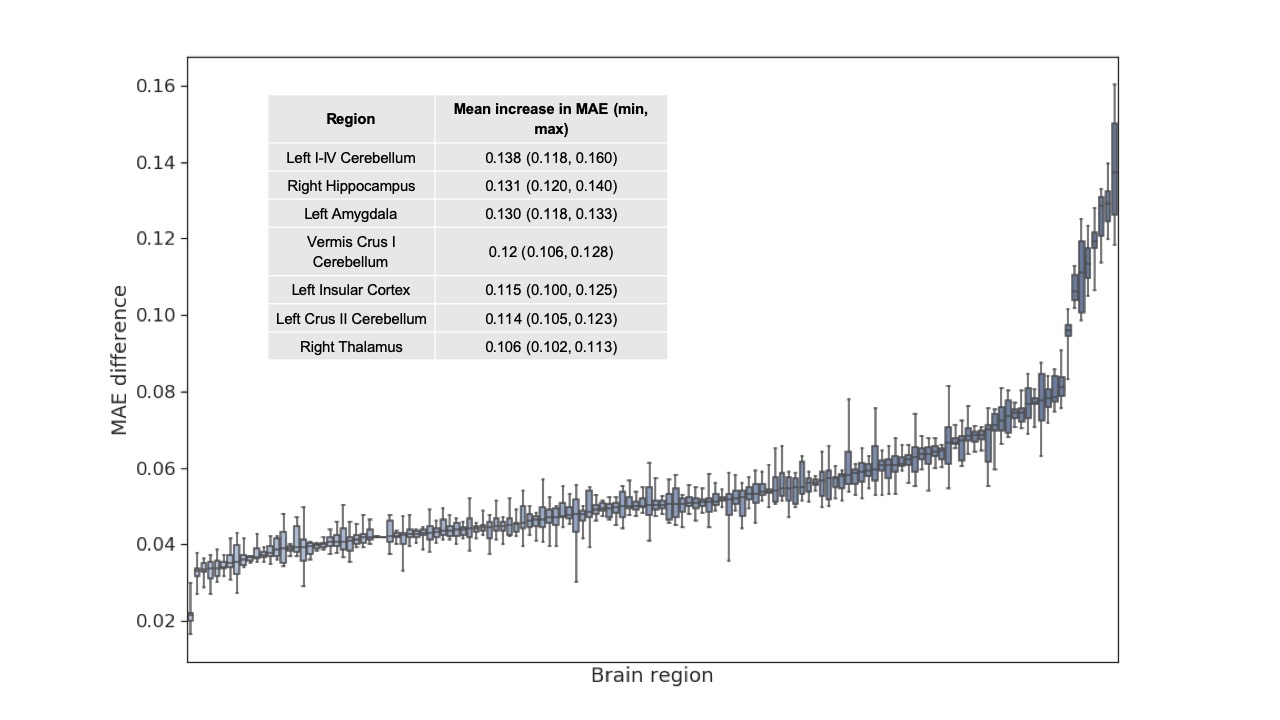
**

**Supplementary Figure 1.** Impact on each of the 139 brain regions (ordered along x-axis) on mean absolute error (MAE) of age prediction accuracy (y-axis). This was calculated using permutation importance, see Methods. It illustrates the effect of removing information contained within the region of interest by replacing it with voxels from the same region in another, random sample, run over the entire test set (N=12,296). The whiskers are the range of samples obtained for 12 repeated trials.


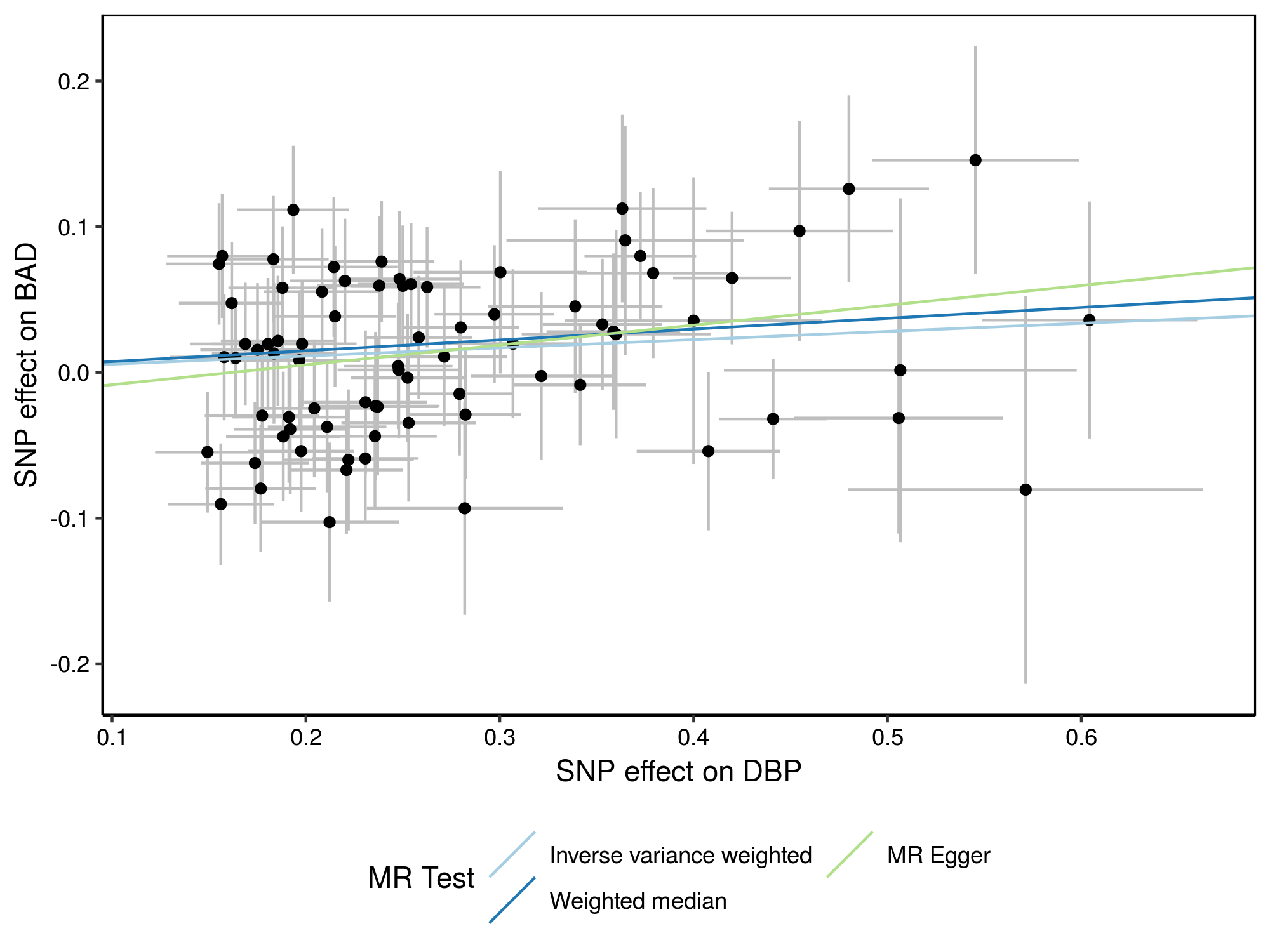


**Supplementary Figure 2.** Mendelian randomisation - a scatter plot indicating the relationship of SNP effects on DBP against SNP effects on brain age difference. Error bars are the range of standard error.

**Supplementary Table 1.** Distribution of healthy and unhealthy individuals defined by their diagnosis status at baseline (healthy: having no recorded diagnosis in hospital episode statistics data in UK Biobank) in the train, validation and test data sets for the machine learning model.

|  | Healthy | Unhealthy | Total |
| --- | --- | --- | --- |
| Train | 3,067 | 0 | 3,067 |
| Validation | 1,071 | 2,891 | 3,962 |
| Test - healthy | 2,057 | 0 | 2,057 |
| Test - unstratified | 1,041 | 11,255 | 12,296 |

**Supplementary Table 2.** Traits that associate with brain age difference, with 2.35 * 10^-5^ (Bonferroni threshold) < p-value < 1.45 * 10^-3^ (FDR threshold). Odds ratios and betas are given per unit standard deviation of brain age difference (3.72 years).

| **Categorical and ordered traits** | **Category** | **Odds ratio (95% CI)** | **p-value** | **Rate of incidence (case/control)** |
| --- | --- | --- | --- | --- |
| Depressive episode | Diagnoses | 3.33 (1.85, 6.01) | 6.04 * 10-05 | 15/12281 |
| Occupational therapy and vocational rehabilitation | Diagnoses | 3.27 (1.75, 6.16) | 2.09 * 10-04 | 13/12283 |
| Diabetic retinopathy | Diagnoses | 2.57 (1.44, 4.59) | 1.34 * 10-03 | 15/12281 |
| Diagnoses - secondary ICD10: Z50.1 Other physical therapy | Diagnoses | 2.02 (1.37, 3) | 4.39 * 10-04 | 33/12263 |
| One or more previous psychiatric episodes with this Health Care Provider | Psychiatric | 1.97 (1.35, 2.87) | 3.83 * 10-04 | 35/10988 |
| Gastro-intestinal haemorrhage | Diagnoses | 1.89 (1.29, 2.76) | 1.11 * 10-03 | 35/12261 |
| Calculus of kidney | Diagnoses | 1.64 (1.25, 2.17) | 4.52 * 10-04 | 67/12229 |
| Epilepsy | Diagnoses | 1.63 (1.23, 2.17) | 7.12 * 10-04 | 64/12232 |
| Personal history of long-term (current) use of anticoagulants | Diagnoses | 1.43 (1.2, 1.72) | 9.72 * 10-05 | 162/12134 |
| Total errors traversing alphanumeric path (trail #2) | Trail making | 1.17 (1.08, 1.28) | 3.22 * 10-04 | 2331 |
| Neuroticism score | Psychosocial factors | 1.07 (1.03, 1.12) | 3.61 * 10-04 | 10355 |
| On hormone replacement therapy | Health and medical history | 0.81 (0.72, 0.91) | 2.37 * 10-04 | 583/1087 |
| Single live birth | Diagnoses | 0.76 (0.67, 0.87) | 8.58 * 10-05 | 546/11750 |
| Second degree perineal laceration during delivery | Diagnoses | 0.64 (0.49, 0.83) | 8.91 * 10-04 | 106/12190 |
| **Continuous traits** | **Category** | **Beta (95% CI)** | **p-value** | **N sample** |
| Visceral adipose tissue volume | Abdominal MRI | 0.06 (0.03, 0.09) | 3.94 * 10-04 | 3627 |
| Duration to complete alphanumeric path (trail #2) | Touchscreen | 0.06 (0.03, 0.08) | 7.12 * 10-05 | 5802 |
| Duration to complete numeric path (trail #1) | Touchscreen | 0.06 (0.03, 0.08) | 1.49 * 10-04 | 5802 |
| Heart rate during PWA | Heart MRI | 0.04 (0.02, 0.06) | 5.55 * 10-04 | 10416 |
| Waist circumference | Physical measures | 0.03 (0.01, 0.05) | 9.58 * 10-04 | 12287 |
| LV stroke volume | Heart MRI | -0.06 (-0.10, -0.02) | 1.36 * 10-03 | 2857 |

**Supplementary Table 3.** Mendelian randomisation results

| **Exposure** | **Method** | **Beta** | **p-value** | **Intercept p-value** | **Heterogeneity p-value** |
| --- | --- | --- | --- | --- | --- |
| Alzheimer’s disease | Inverse variant weighted | -0.02 | 0.36 | N/A | 0.83 |
|  | Weighted mean | -0.01 | 0.67 | N/A | N/A |
|  | MR Egger | -0.02 | 0.60 | 0.95 | 0.80 |
| Diabetes | Inverse variant weighted | 0.03 | 0.57 | N/A | 0.64 |
|  | Weighted mean | 0.10 | 0.21 | N/A | N/A |
|  | MR Egger | 0.18 | 0.18 | 0.23 | 0.66 |
| Diastolic blood pressure | Inverse variant weighted | 0.06 | 0.01 | N/A | 0.14 |
|  | Weighted mean | 0.07 | 0.02 | N/A | N/A |
|  | MR Egger | 0.14 | 0.05 | 0.23 | 0.15 |
| Systolic blood pressure | Inverse variant weighted | 0.01 | 0.22 | N/A | 0.08 |
|  | Weighted mean | 0.03 | 0.08 | N/A | N/A |
|  | MR Egger | 0.05 | 0.34 | 0.54 | 0.07 |
| Pulse pressure | Inverse variant weighted | -0.03 | 0.04 | N/A | 0.003 |
|  | Weighted mean | -0.04 | 0.28 | N/A | N/A |
|  | MR Egger | -0.17 | 0.317 | 0.37 | 0.003 |
